# Identification and Characterization of an Antennae-Specific Aldehyde Oxidase from the Navel Orangeworm

**DOI:** 10.1371/journal.pone.0067794

**Published:** 2013-06-24

**Authors:** Young-Moo Choo, Julien Pelletier, Elizabeth Atungulu, Walter S. Leal

**Affiliations:** Honorary Maeda-Duffey Laboratory, University of California Davis, Davis, California, United States of America; AgroParisTech, France

## Abstract

Antennae-specific odorant-degrading enzymes (ODEs) are postulated to inactivate odorant molecules after they convey their signal. Different classes of insect ODEs are specific to esters, alcohols, and aldehydes – the major functional groups of female-produced, hydrophobic sex pheromones from moth species. Esterases that rapidly inactive acetate and other esters have been well-studied, but less is known about aldehyde oxidases (AOXs). Here we report cloning of an aldehyde oxidase, AtraAOX2, from the antennae of the navel orangeworm (NOW), *Amyelois transitella*, and the first activity characterization of a recombinant insect AOX. *AtraAOX2* gene spans 3,813 bp and encodes a protein with 1,270 amino acid residues. AtraAOX2 cDNA was expressed in baculovirus-infected insect Sf21 cells as a ≈280 kDa homodimer with 140 kDa subunits. Recombinant AtraAOX2 degraded Z11Z13–16Ald and plant volatile aldehydes as substrates. However, as expected for aldehyde oxidases, recombinant AtraAOX2 did not show specificity for Z11Z13–16Ald, the main constituent of the sex pheromone, but showed high activity for plant volatile aldehydes. Our data suggest AtraAOX2 might be involved in degradation of a diversity of aldehydes including sex pheromones, plant-derived semiochemicals, and chemical cues for oviposition sites. Additionally, AtraAOX2 could protect the insect's olfactory system from xenobiotics, including pesticides that might reach the sensillar lymph surrounding the olfactory receptor neurons.

## Introduction

In insects, the olfactory system plays an important role in communicating the availability of food sources, habitats, and oviposition sites as well as in locating mates. Odorant receptors (ORs), ionotropic receptors (IRs), sensory neuron membrane proteins (SNMPs), odorant-binding proteins (OBPs), and odorant-degrading enzymes (ODEs) in hair-like sensilla on antennae are crucial for reception of odorants (chemical signals). The roles of the three major olfactory proteins, i.e., ORs, OBPs and ODEs, are well discussed elsewhere [1]. In short, OBPs, including pheromone-binding proteins (PBPs), deliver odorants entering the sensillar lymph to ORs, and at the end of the journey, ODEs play a pivotal role in degrading stray odorants in the peripheral space that could interfere with the fidelity and sensitivity of the insect's olfactory system [1], [2]. During flight male moths need to rapidly inactivate stray odor molecules and reset the olfactory system on a millisecond timescale [3], [4]. Their response to sex pheromones can be enhanced or inhibited by plant-derived compounds [5]. Thus, ODEs or pheromone-degrading enzymes (PDEs) could be potential molecular targets for pest control because they play an important role in degrading or inactivating stray odor molecules around the receptor lymph and in the internal cellular space [2]. Although antennae-specific esterases, which degrade sex pheromones with an ester moiety have been well documented in the literature [4], [6], [7], the role of other ODEs are still poorly understood [2].

Long-chain, unsaturated alcohol and aldehyde compounds are common female-produced sex pheromones [8]–[12]. Rybczynski and his colleague identified antennae-specific aldehyde oxidases (AOXs) in *Manduca sexta, Antheraea polyphemus* and *Bombyx mori*, which degraded aldehydic sex pheromone such as bombykal [13], [14]. *M. sexta* AOX from male and female antennae showed activity against bombykal and plant volatile aldehydes [13]. However, aldehyde oxidases are well-known cytosolic enzymes that lack signal peptide. The only noticeable research on AOXs over the past decade is the cloning and molecular characterization of antennae-specific aldehyde oxidase genes from *B. mori* (*BmorAOX1* and *BmorAOX2*) [15], but expression of the enzymes encoded by these genes and elucidation of their physiological roles remain as areas for future research.

*Spodoptera littoralis* antennal esterase 10 (SICXE10), which occurs intracellularly in both male and female antennae, was first expressed in baculovirus-infected cells and functionally characterized. SICXE10 was more sensitive to green leaf volatiles than to a sex pheromone, suggesting that it could degrade a high background of various plant volatiles and detoxify xenobiotics, such as insecticide molecules entering the sensilla [16]. Recent studies indicated that plant volatiles interfere with the reception of sex pheromone in moths and beetles [17]–[22]. Thus, reducing plant volatile signals might enhance reception of pheromones. Additionally, non-olfactory aldehyde oxidase and esterase in mosquitoes have been well documented as xenobiotic-degrading enzymes [23], [24], and plant volatile aldehydes have been reported as potent insecticides [25], [26]. For instance, acetaldehyde is so toxic that it must be degraded in insects for their survival in the environment [27], [28]. While non-olfactory aldehyde oxidases in insects have been well studied as one important factor for survival, their precise physiological role in the olfaction, other than the suggested role of pheromone degradation, are unknown.

Here we report the isolation of an antennae-specific aldehyde oxidase from the navel orangeworm (NOW), *Amyelois transitella* and its full characterization using recombinant enzyme purified from baculovirus-infected insect cells. We demonstrate its expression pattern, enzyme property, and enzyme activity on the sex pheromone and plant volatile aldehyde compounds, including aldehyde-containing xenobiotics.

## Results and Discussion

### Identification of an Aldehyde Oxidase in the Antennae of the Navel Orangeworm

**Figure 1:**
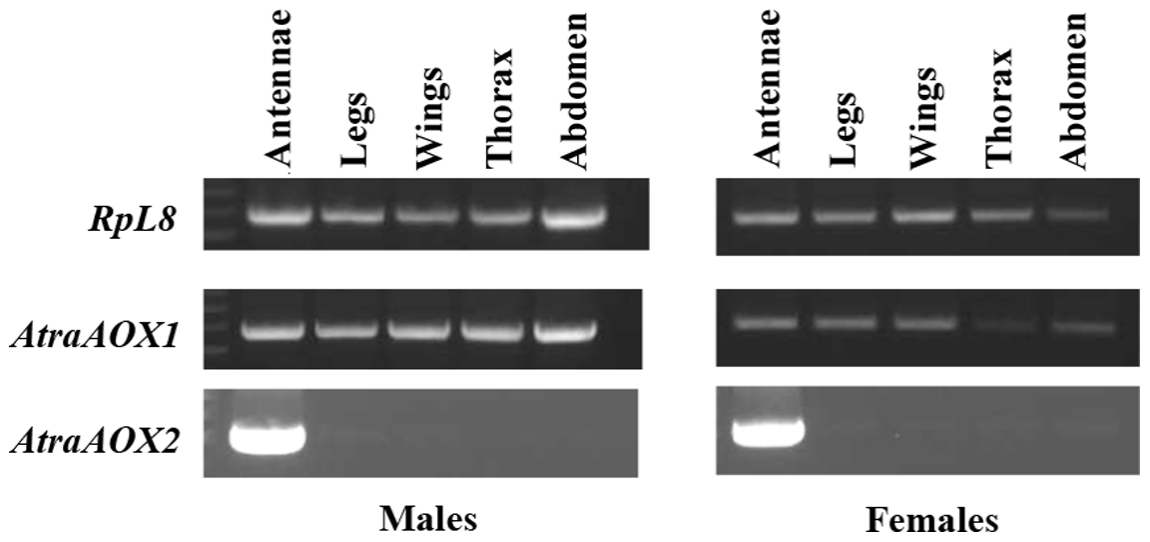
Transcription profiles of *AtraAOX1* and *AtraAOX2* by RT-PCR. *AtraAOX1* gene product was detected in all male and female tissues (antennae, legs, wings, thorax, and abdomen), whereas *AtraAOX2* gene was specifically transcribed in male and female antennae.

**Figure 2:**
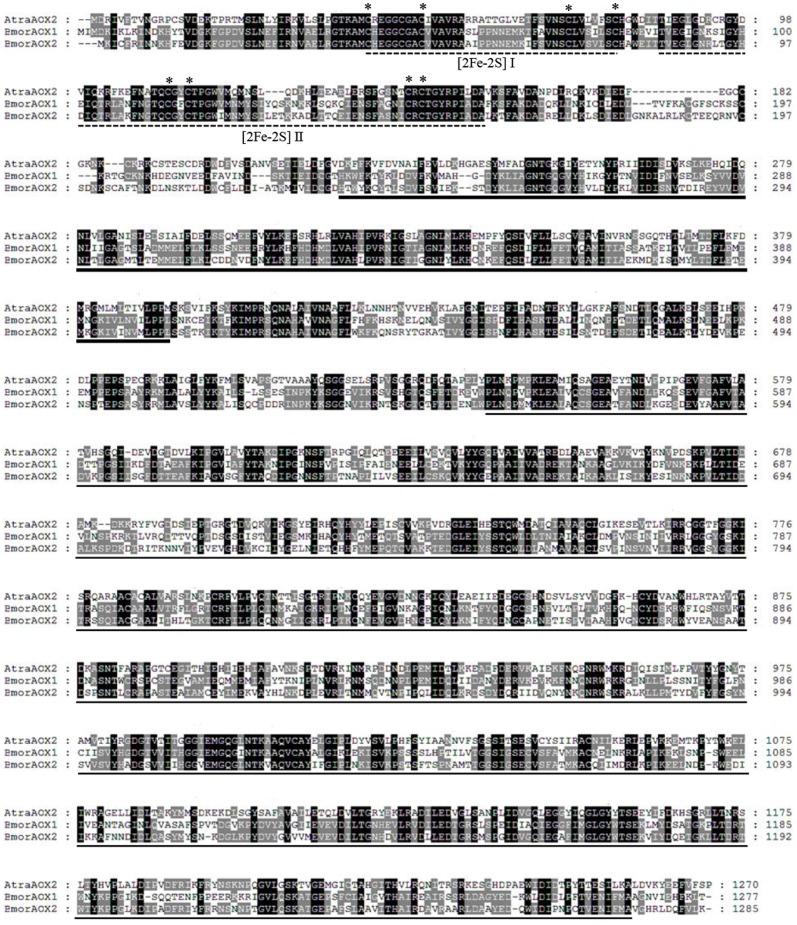
Amino acid sequence alignment of AtraAOX2 and known antennal BmorAOX1 and BmorAOX2. Identical residues are shaded in black and similar residues are shaded in gray. The two [2Fe-2S] redox centers are indicated in dotted line. The characteristic cysteine residues involved in the formation of the [2Fe-2S] centers are indicated by asterisk. The FAD-binding domain is underline in bold. The molybdenum (MoCo) cofactor and substrate-binding domains are underlined. Alignment of amino acid sequences were carried out with GeneDoc 2.7.0 software [61].

To explore the physiological role of aldehyde oxidases in antennae, we isolated two aldehyde oxidases from NOW antennae. We then performed RT-PCR analysis to determine whether our selected *AtraAOXs* are specifically expressed in male or female antennae. *AtraAOX2* was specifically expressed in antennae of male and female moths, whereas *AtraAOX1* was expressed in non-olfactory tissues such as legs, wings, thorax, abdomen, and male and female antennae (Fig. 1). Based on its transcription profile, *AtraAOX2* could be potentially involved in some specific olfactory function. AtraAOX2 (GenBank, KC952900) consists of 1270 amino acids lacking signal peptide (SignalP 4.1 Server; http://www.cbs.dtu.dk/services/SignalP/) [29]. Database search showed that this protein contains common features consistent with antennae-specific aldehyde oxidases in *B. mori* [15]. Based on [30], AtraAOX2 contains two putative iron-sulfur (2Fe-2S) redox center, flavin-containing region (FAD-binding domain), molybdenum cofactor-binding site and eight cysteine residues involved in the coordination with iron ions (Fig. 2).

### Expression and Purification of Recombinant AtraAOX2

**Figure 3:**
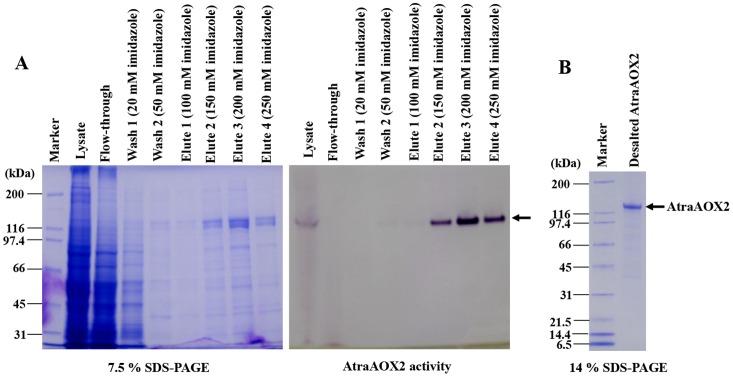
Expression and purification of recombinant AtraAOX2. (A) 6×His-tagged AtraAOX2 was purified on Ni-NTA superflow under native conditions. Purified fractions were analyzed by 7.5% SDS-PAGE (left) and aldehyde-oxidase activity assay using 1 mM benzaldehyde as a substrate (right). (B) Eluted fractions were desalted and concentrated using Ultracel 100 K (100 kDa cut-off), and desalted AtraAOX2 (2 µg) was analyzed by 14% SDS-PAGE. The arrow on the right indicates the position of AtraAOX2.

To assess the physiological role of AtraAOX2, we attempted to express AtraAOX2 first in bacterial and then with a baculovirus expression system. We employed two bacterial expression systems, different host cells, and multiple conditions, but were unable to express this enzyme (see Materials and Methods). In our first attempts with a baculovirus expression system, recombinant AtraAOX2 was detected in the cell lysate, but most of the sample was lost during several purification steps given the low levels of expression. Subsequently, we succeeded in purifying recombinant AtraAOX2 by using 6×His-tag system in the C-terminal end. The recombinant protein was analyzed by SDS-PAGE and MTT-based gel stain (zymogram) (Fig. 3A). In order to fully characterize AtraAOX2, the eluted sample was further purified using Ultracel 100 K. The purified recombinant enzyme showed a single band with 140 kDa molecular weight (Fig. 3B).

**Figure 4:**
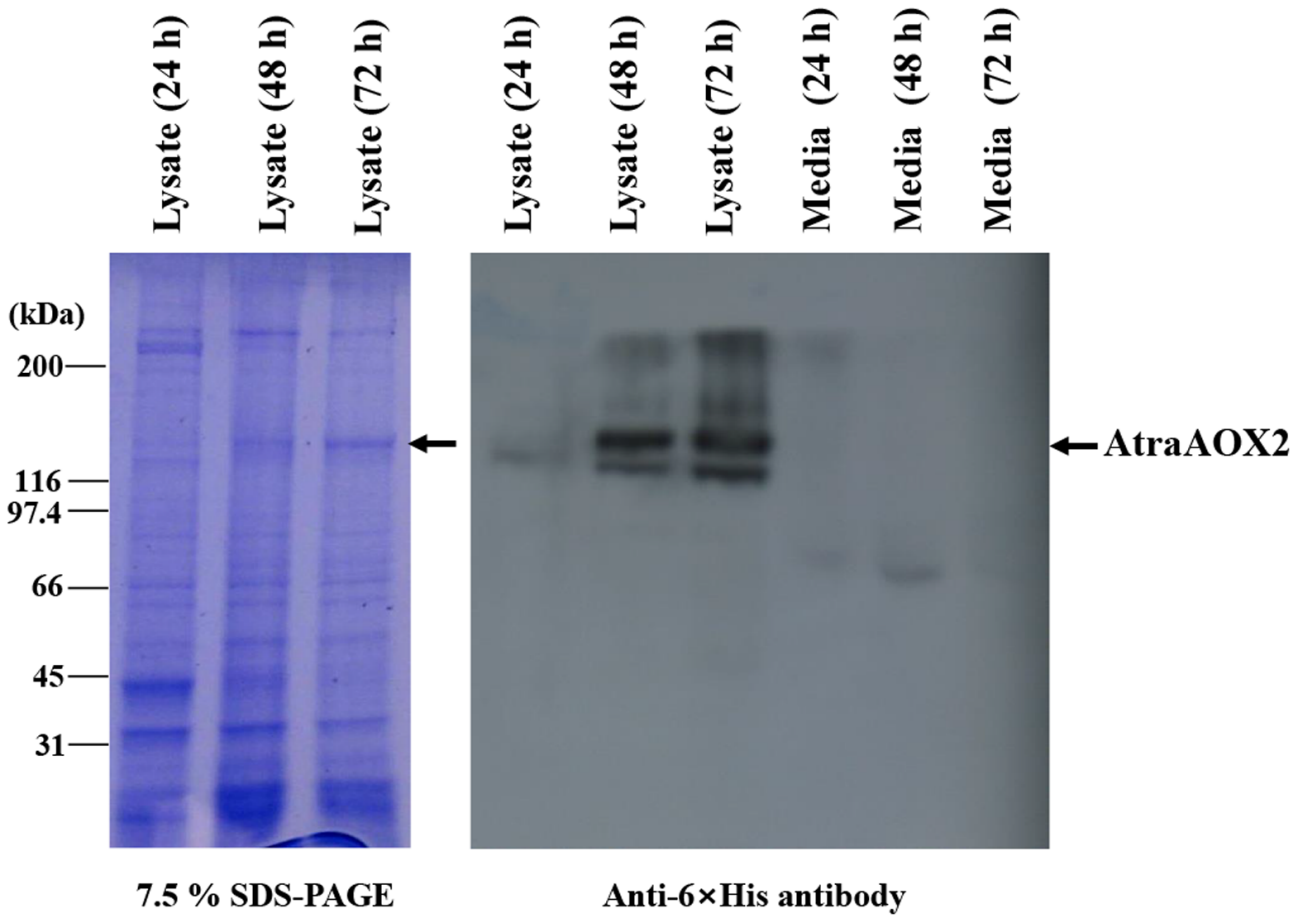
Expression of the recombinant AtraAOX2 in baculovirus-infected insect cells. Cells were infected with the recombinant AcNPV-AtraAOX2 at an MOI (multiplicity of infection) of 10 PFU (plaque forming unit) per cell and collected at 1-, 2-, and 3-day post-infection., respectively. Total cellular lysates and media were analyzed by 7.5% SDS-PAGE (left) and Western blots with an anti-6×Histidine antibody (right).

An earlier study by Rybczynski et al. reported that aldehyde oxidase in antennae of the tobacco hornworm moth, *M. sexta*, degraded the aldehydic pheromone bombykal in the sensillar lymph, indicating that the enzyme is secreted around the receptor lymph [13]. To investigate the secretion of AtraAOX2, protein expressed in Sf21 cell infected with the recombinant virus was analyzed by SDS-PAGE and western blot (Fig. 4). The protein in recombinant virus-infected Sf21 cells was detected in the cellular lysate but not in the culture media, indicating that AtraAOX2 is a cytosolic protein. These types of enzymes have no signal peptides that are required to enter the secretory pathway, and there is no evidence for a membrane translocation mechanism [1].

### Determination of native molecular weight and enzyme activity

**Figure 5:**
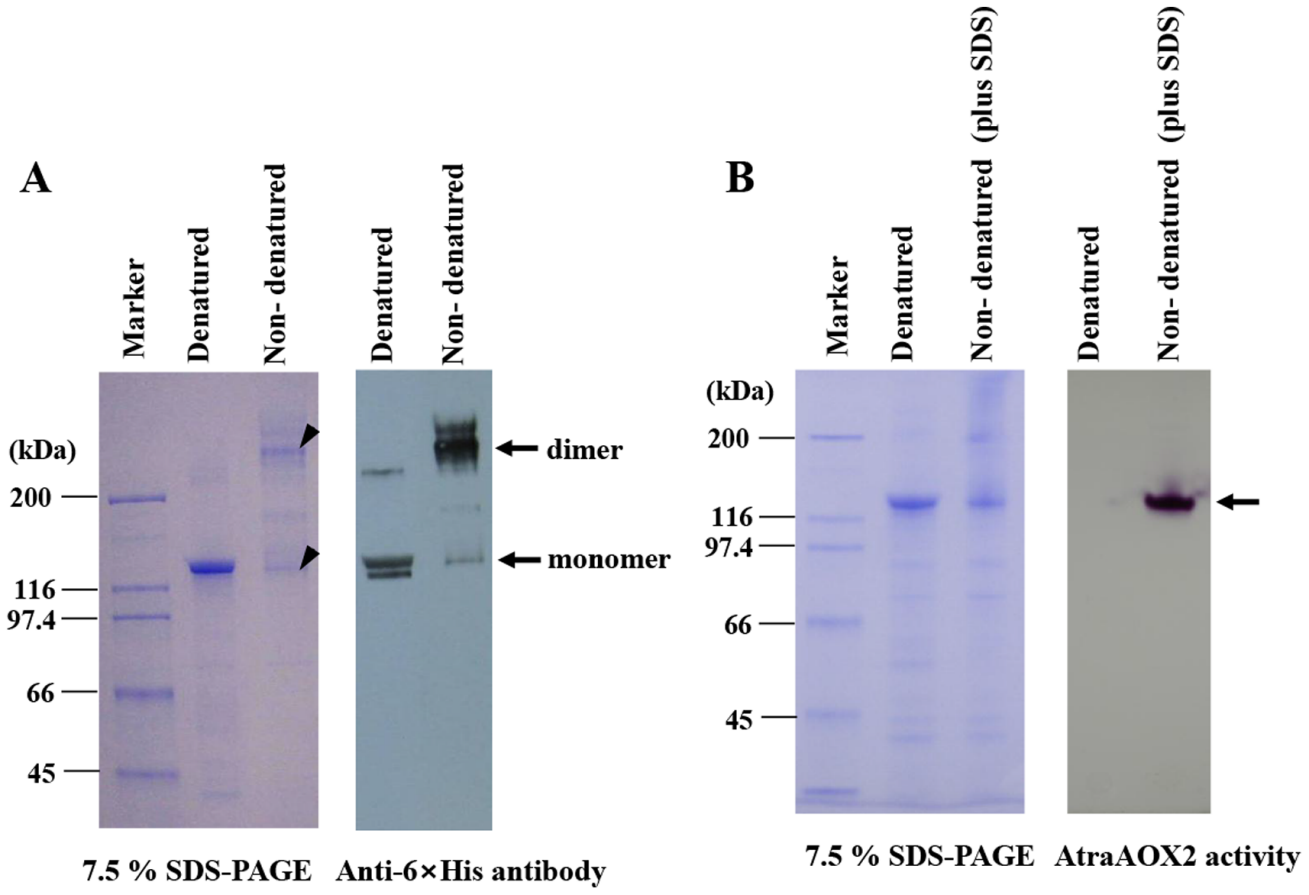
Determination of native molecular weight for monomer/dimer AtraAOX2. (A) Monomer/dimer AtraAOX2 (2 µg) were analyzed by 7.5% SDS-PAGE (left) and Western blots with a 6×His-tag antibody (right). Denatured AtraAOX2 (2 µg), dissolved in a sample buffer containing SDS and β-ME, was boiled. Non-denatured AtraAOX2 (2 µg), dissolved in a sample buffer without SDS and β-ME, was not boiled. The arrowhead indicates the position of monomer and dimer of AtraAOX2. (B) Monomer AtraAOX2 activity was analyzed by 7.5% SDS-PAGE (left) and aldehyde-oxidase activity using 1 mM benzaldehyde as a substrate (right). Denatured AtraAOX2 was boiled with SDS and β□ME, and non-denatured AtraAOX2 was not boiled but SDS was added.

Aldehyde oxidases existed naturally as a homodimer or heterodimer composed of two subunits in many organisms [13], [31]–[33]. To determine the native molecular mass of AtraAOX2, the recombinant protein was analyzed by SDS-PAGE and Western blot (Fig. 5A). Western blot analysis indicated that recombinant AtraAOX2 was expressed as a ≈280 kDa homodimer composed of 140 kDa subunits. Dimer formation of AOXs may be necessary for catalytic activity. A monomer of rat strain AOX showed low activity with their substrate [34]. However, a monomer of AtraAOX2 separated by SDS still showed strong activity, indicating that a monomeric AtraAOX2 has a rather stable and active structure (Fig. 5B). These findings indicate that the dimer is linked by non-covalent bonds, as previously suggested for *M. sexta* aldehyde oxidase (AOX) [13].

**Figure 6:**
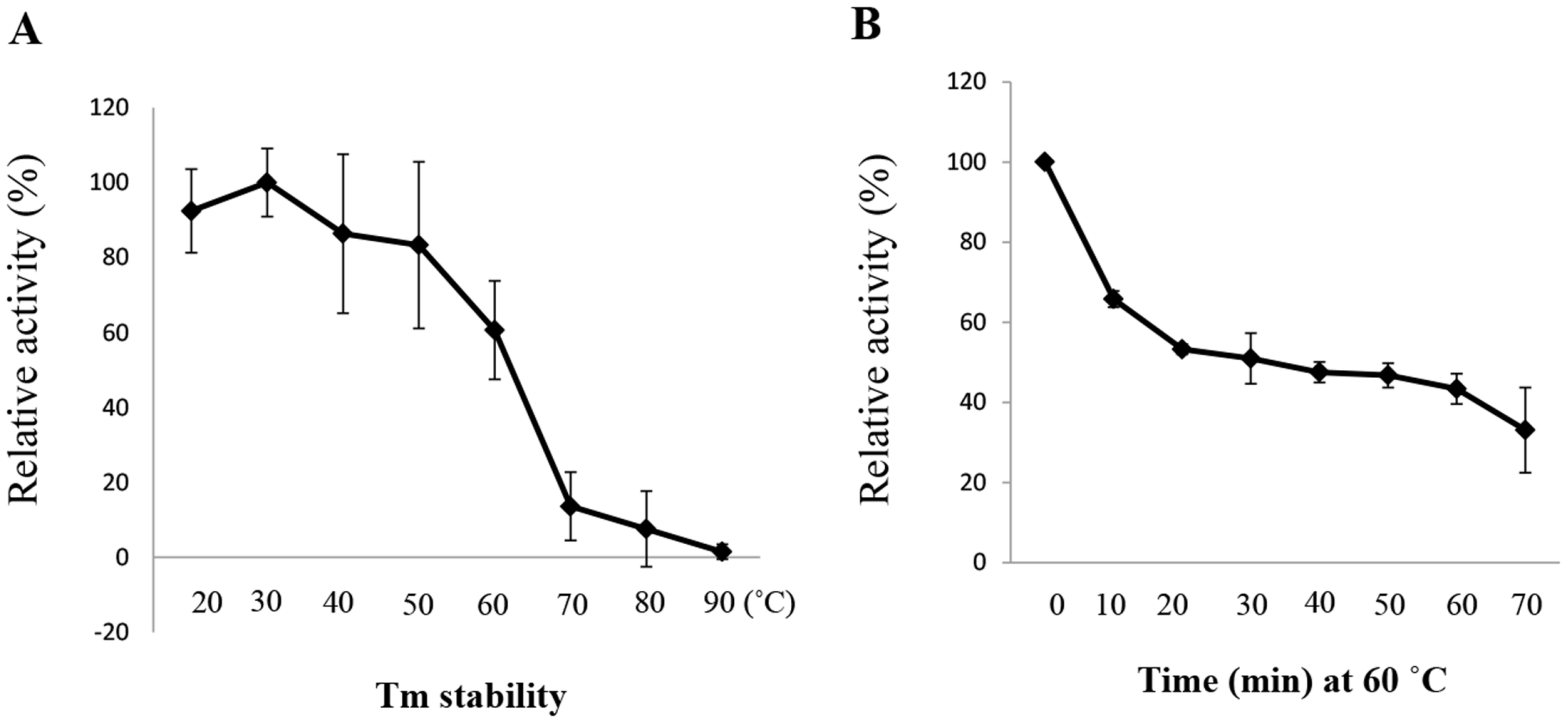
Thermal stability and heat inactivation at 60°C. (A) For thermal stability, AtraAOX2 (1 µg) was incubated at different temperatures ranging from 20 to 90°C for 10 min at pH 8 without a substrate, enzyme activity was determined at 30°C using 1 mM benzaldehyde as a substrate, and then quenched with 10% acetic acid. The reduction of MTT (at 570 nm) was measured spectrophotometrically. (B) AtraAOX2 (1 µg) were preheated at 60°C without a substrate. Samples were removed at each time point for 70 min, chilled on ice, and then enzyme activity was determined at 30°C using 1 mM benzaldehyde as a substrate. Data are expressed as the mean of thee assays (n = 3).

The thermostability of recombinant AtraAOX2 was determined by measuring reduction of thiazolyl blue tetrazolium bromide (3-(4,5-Dimethyl-2-thiazolyl)-2,5-diphenyl-2H-tetrazolium bromide, MTT; 570 nm) at different temperatures. AtraAOX2 was relatively stable from 20 to 60°C but activity dropped dramatically at 70°C (Fig. 6A).

Heat inactivation of aldehyde oxidases from several strains of *Drosophila melanogaster* indicated that there were two forms of AOXs, one rapidly and the other slowly inactivated [35], [36]. Under the same conditions, heat inactivation profile of *M. sexta* AOX showed a monophasic decline in activity [13]. AtraAOX2 preheated at 60°C, showed biphasic inactivation (Fig. 6B), with a rapid decrease from 0 to 10 min, and monotonous decrease thereafter, but the estimated half-life (33 min) was similar to that reported for *M. sexta* AOX [13].

**Figure 7:**
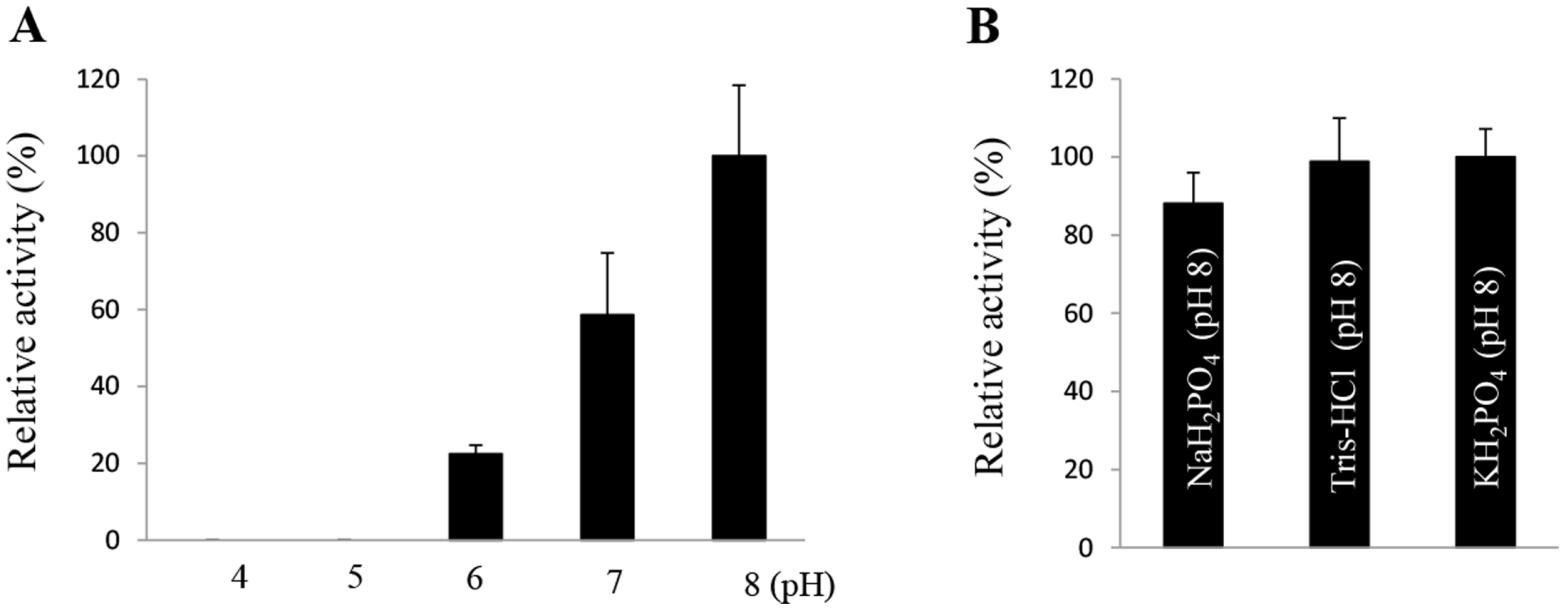
pH profile and optimal buffer for AtraAOX2. (A) Enzymatic activity on 1 mM benzaldehyde was assayed at various pH values from 4 to 8 at 30°C. Enzyme activity was determined at 30°C using 1 mM benzaldehyde as a substrate and quenched with 10% acetic acid. The reduction of MTT (at 570 nm) was measured spectrophotometrically. Higher pH values could not be determined because MTT resulted in rapid nonenzymatic reduction. (B) To determine optimal buffer, AtraAOX2 activity was assayed at 30°C using 1 mM benzaldehyde as a substrate, with each of the test buffer at 100 mM (pH 8). Data are expressed as the mean of thee assays (n = 3).

To determine the optimal pH of AtraAOX2, we measured the activity of the enzyme at various pH values from 4 to 8. AtraAOX2 had an optimal pH value of 8 (Fig. 7A) within the tested range. Activity at higher pH could not be determined because of the rapid non-enzymatic reduction of MTT [13]. Additionally, enzyme activity was tested with three different buffers at pH 8, but no significant difference was observed (Fig. 7B).

### The Role of Antennal-Specific AtraAOX2 in Olfaction

**Figure 8:**
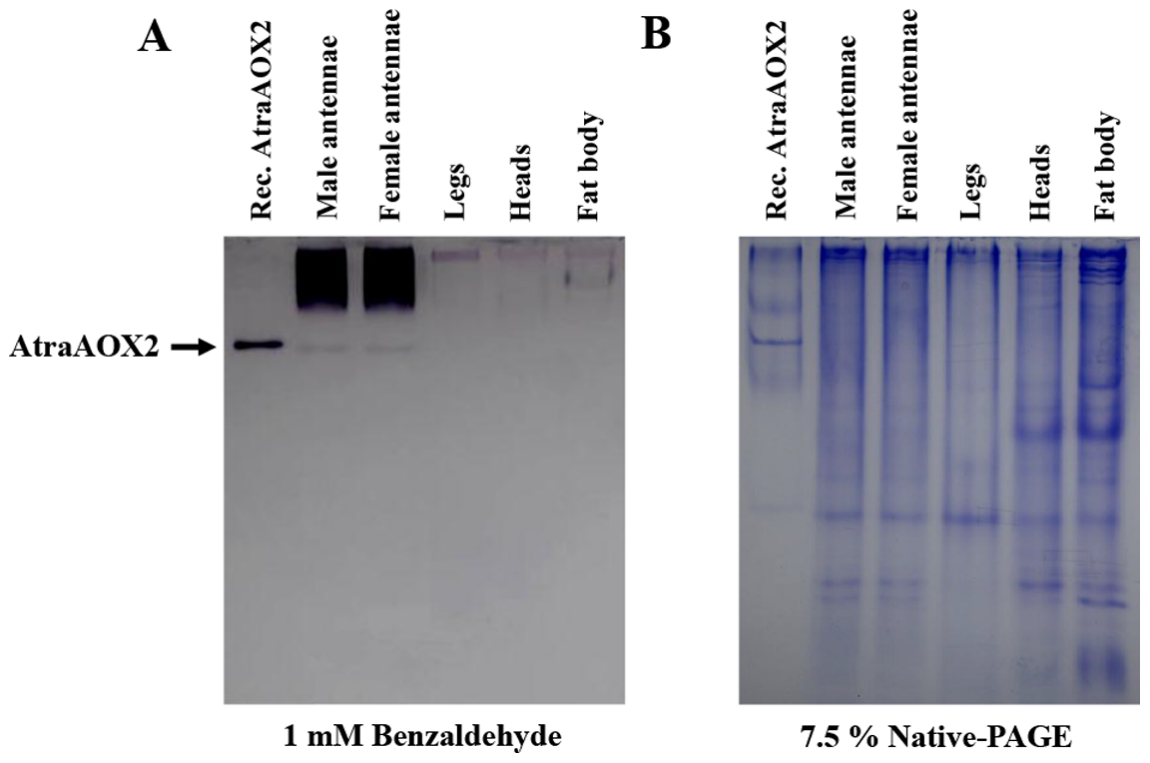
Tissue distribution in male and female.

**Figure 9:**
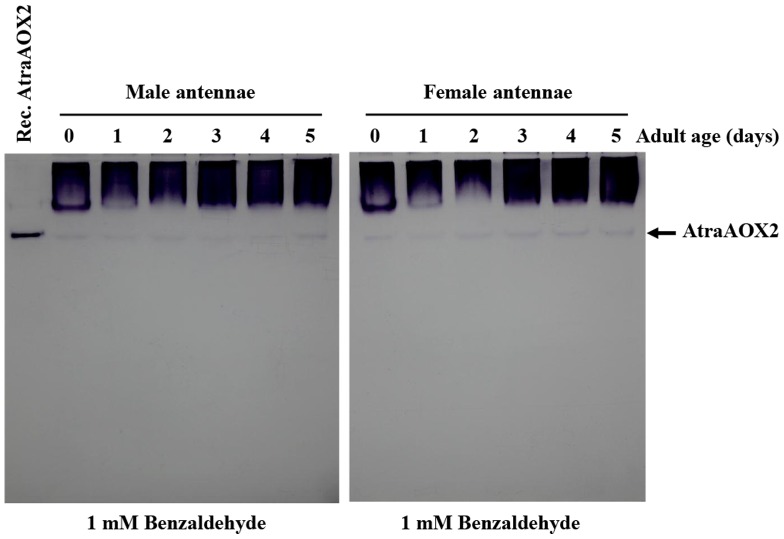
Protein expression profile in male and female antennae. Protein samples (30 µg equal concentration/lane) were extracted from the antennae of *A. transitella* male and female adults, and analyzed by 7.5% Native-PAGE as described above. The arrow on the right indicates the position of AtraAOX2. Recombinant AtraAOX2 showed a slightly higher position than native AtrAOX2 because of 6×His-tagged.

To investigate the potential role of AtraAOX2 in *A. transitella*, we determined the expression pattern from various tissues and ages of NOW adults with zymograms. As observed from the RT-PCR data (Fig. 1), AtraAOX2 was expressed only in male and female antennae (Fig. 8). Interestingly, the expression level of AtraAOX2 was nearly constant during the adult stage (Fig. 9) thus suggesting that this enzyme could ensure a vital role throughout the entire life of male and female adults.

**Figure 10:**
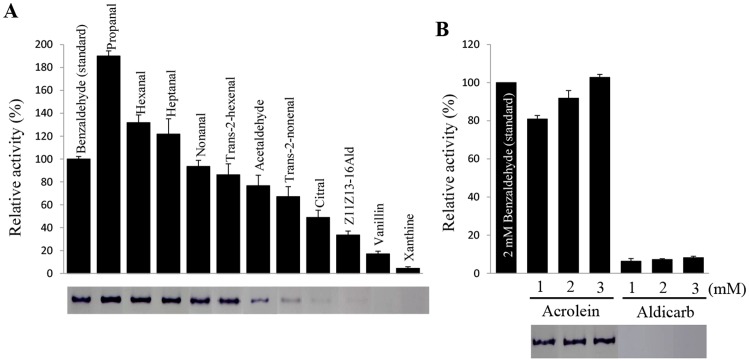
Oxidation of various substrates by AtraAOX2. (A) The oxidation of 2 mM substrates by AtraAOX2 (1 µg) was determined at 30°C and quenched with 10% acetic acid. The reduction of MTT (at 570 nm) was measured spectrophotometrically. Substrates lacking AtraAOX2 were used as blank control in order to prevent non-enzymatic background activity. To confirm AtraAOX2 activity, a zymogram was performed using 2 mM of each substrate on 7.5% Native-PAGE, respectively. (B) The oxidation of dose-dependent pesticide compounds as substrate by AtraAOX2 (1 µg) was determined as described above.

In male moths, pheromone-degrading enzymes (PDEs) could have critical role in terminating chemical signal during flight. Pheromone-degrading esterases that rapidly degrade sex pheromones with an ester functional group have been well-studied [4], [6], [7], [37]–[39]. Antennal AOXs in *M. sexta, A. polyphemus* and *B. mori* were studied for their role in degradation of the sex pheromone, bombykal, as well as non-pheromone aldehydes derived from host plants [13], but genes encoding these proteins have not been identified to date. Although genes from two *AOXs* from *B. mori, BmorAOX1* and *BmorAOX2*, have been isolated and characterized at the molecular level, the physiological function(s) of the proteins encoded by these genes are yet to be studied [15]. To determine whether recombinant AtraAOX2 can degrade the main NOW sex pheromone Z11Z13–16Ald and plant volatile aldehydes, activity of AtraAOX2 on various substrates was measured spectrophotometrically and confirmed by zymograms (Fig. 10A). AtraAOX2 showed strong activity on aldehyde substrates derived from plants, but weak activity on Z11Z13–16Ald. This could be in part due to the lower solubility of moth sex pheromones as compared to the other substrates tested. However, attempts to improve solubility of Z11Z13–16Ald with dimethyl sulfoxide (DMSO) were unrewarding, although the low amounts of solvent added did not affect enzyme activity (data not shown). Recently, Durand et al. suggested that the main physiological function of *S. littoralis* esterase 10 (SICXE 10) is degradation of plant volatile compounds rather than sex pheromone [16]. We propose that degradation of plant volatile by AtraAOX2 might be important for pheromone reception in the navel orangeworm considering that high levels of plant odorants might attenuate reception of sex pheromones as recently reported [22].

In mammals and in the mosquito *Culex quinquefasciatus*, the role of AOXs is well known as they play a critical role in detoxifying several environmental pollutants (xenobiotics) such as pesticides [23], [24], [40], [41]. Acetaldehyde, an air pollutant, is naturally produced in leaves and fruits of plants [42], [43]. It is highly toxic and must be degraded into non-toxic products [27], [28]. Metabolism of acetaldehyde by cytosolic oxidizing enzymes is important for survival of *D. melanogaster* larvae and adult [44]–[46]. Volatile aldehydes such as propanal and (*E*)-2-hexenal were also reported as having insecticidal activity in fumigation assay [25], [26], and green leaf volatiles, including α,β-unsaturated aldehyde derivatives, are involved in plant-defense responses against herbivores and pathogens [47]–[50]. To determine whether AtraAOX2 plays a role in degrading pesticides, we investigated AtraAOX2 activity on the herbicide acrolein ( = 2-propenal, acrylaldehyde), and the insecticide, aldicarb ( = 2-methyl-2-(methylthio)propanal *O*-(*N*-methylcarbamoyl)oxime). Although AtraAOX2 did not degrade the carbamate insecticide aldicarb, it was active on the pesticide with an aldehyde functional group (acrolein) (Fig. 10B). These results indicate that AtraAOX2 in antennae of NOW also functions as a xenobiotic-degrading enzyme. Previous work with a cytosolic glutathione-*S*-transferase from the antenna of *M. sexta* (GST-msolf1), showed that it too plays a dual role by degrading xenobiotic and odorant substrates [51]. Taken together, we strongly suggest that AtraAOX2 could be involved in detoxifying plant-derived toxic aldehydes and aldehyde-containing pesticides.

Several inhibitors of aldehyde oxidases have been well documented for mammals, plant, and insect AOXs [13], [52]–[56]. We also tested the effect of various inhibitors on recombinant AtraAOX2 using propanal as a substrate (Table 1). Hydroquinone and sodium deoxycholate had no inhibitory effect, but rotenone, Triton X-100, potassium cyanide, digitonin, isopropanol, menadione, ethanol, β-estradiol, p-hydroxymercuribenzoate, and sodium azide had moderate inhibitory effects (about 10∼50%). On the other hand, β-ecdysone, methanol, quinacrine, antimycin A, and dinitrophenol had high inhibitory effects (about 55∼70%).

**Table 1:** Effect of various inhibitors on propanal oxidation by AtraAOX2.

Inhibitor	Concentration	Activity (%)
Control		100
Hydroquinone	10 mM	105.8
Na deoxycholate	240 µM	102.9
Rotenone	10 µM	89.3
Triton X-100	0.01%	71.8
KCN	1 mM	71.8
Digitonin	125 µM	69.9
Isopropanol	2%	67.9
Menadione	100 µM	63.1
Ethanol	2%	62.1
β-estradiol	50 µM	60.2
p-hydroxymercuribenzoate	50 µM	55.3
NaN_3_	5 mM	52.4
Antimycin A	10 µM	45.6
Dinitrophenol	50 µM	41.7
Quinacrine	1 mM	34.9
Methanol	2%	33.9
β-ecdysone	50 µM	30.1

AtraAOX2 (1 µg) was pre-incubated with inhibitors for 3 min at 30°C before reaction was started with 2 mM propanal as a substrate. Enzyme activity was determined at 30°C and quenched with 10% acetic acid. The reduction of MTT (at 570 nm) was measured spectrophotometrically. Data are expressed as the mean of thee assays (n = 3).

## Conclusions

Our results suggest that an antennae-specific aldehyde oxidase from the navel orangeworm, AtraAOX2, might be involved in degradation of host plant volatile compounds and pheromone. These functions might be important for lowering the high “background noise” generated by plant volatiles when detecting pheromones, as well as for the direct reception of plant kairomones. Additionally, we suggest that this enzyme help protects the olfactory system from plant-derived xenobiotics and aldehyde-containing pesticides that might reach the sensillar lymph.

## Materials and Methods

### Insects

The *A. transitella* moths used in this study were from a colony kept in our laboratory at the University of California-Davis, which was initiated with moths provided by Charles Burks, United States Department of Agriculture-Agricultural Research Service (Parlier, CA, USA) [57]. Larvae were reared in 1.5 L glass jars on a wheat bran, brewer's yeast, honey, and Vanderzant vitamins (Sigma-Aldrich, St. Louis, MO, USA) diet [58]. Jars were filled with 300 ml of diet to which ca. 300 eggs were added. Cultures were maintained in growth chambers (Percival Scientific, Perry, IA, USA) at 27°C, 70%RH, and a 16∶8 h (light:dark) photo regime. For colony maintenance newly emerged male and female moths were separated (ca. 50 males and ca. 50 females) into 12 × 12 × 5 cm plastic containers (Rubbermaid, Fairlawn, OH, USA) and lined at the bottom with one layer of moist paper towels and lined at the top with one layer of dry paper towels (Thirsty Ultra Absorbent, 27.9 × 27.9 cm; Safeway, Phoenix, AZ, USA) and left in rearing conditions for 72 h. After 72 h the top sheet containing red fertilized eggs was washed in a 10% formaldehyde solution for 15 min and rinsed with double-distilled water and allowed to air dry overnight. These eggs were then used for mass colony rearing.

### Transcription profiles by RT-PCR

Total RNA of different tissue samples (antennae, legs, wings, thorax, abdomen) from males and females NOW was extracted with TRIzol reagent (Invitrogen, Carlsbad, CA), treated with DNase I and cDNAs were prepared with SuperScript II reverse transcriptase (Invitrogen), following manufacturer's instructions. Gene specific primers for AtraAOX1 (AtraAOX1F: 5′- CGAGCGAGGCGCGCCACGACC-3′; AtraAOX1R: 5′- GCTTCTGTGAGGTGTTTGTCCTG-3′) and AtraAOX2 (AtraAOX2F: 5′- TCAGTCTTGCAGACTCACCCCCTG-3′; AtraAOX2R: 5′- GCATACTTAACCGCTTCGCGTTT-3′) were designed for tissue-specificity study. Ribosomal protein L8 encoding gene was used as a control of cDNA integrity (RpL8F: 5′- GAGTCATCCGAGCTCARMGNAARGG-3′; RpL8R: 5′- CCAGCAGTTTCGCTTNACYTTRTA-3′). PCR was performed with GoTaq DNA polymerase (Promega, Madison, WI) and 1 µl cDNA, according to manufacturer's instructions. PCR products were loaded onto 1.5% agarose gel.

### Cloning AOXs from the antennae of NOW

For isolation of AOXs in the antennae of the NOW, a degenerate PCR cloning strategy was used. A pair of degenerate primers were designed based on conserved regions of antennal AOXs from the silkworm moth [15] (AtraAOXdegF: 5′-GARGGIGGITGYGGIGYITGYRT-3′; AtraAOX2degR: 5′-ATIGGICKRTAICCIGTRCAICKRCA-3′) and used in PCR to amplify a partial fragment of AOX from antennal cDNA, prepared as described above. Amplification was performed with Pfu Ultra II polymerase (Agilent Technologies, Santa Clara, CA) and 1 µl cDNA, according to manufacturer's instructions. Two different AOX amplicons were isolated, purified, sequenced (Davis Sequencing Inc. Davis, CA) and named AtraAOX1 and AtraAOX2. A larger fragment of AtraAOX2 was obtained by PCR, by combining a gene specific primer (AtraAOX2F: 5′-CAGGACAAACACCTCACAGAAGC-3′), designed within the partial fragment, with another degenerate primer located downstream on another AOX conserved region (AtraAOX2degR2: 5′-ACCTTGACCCATTTCWAYWCCACC-3′). Amplification was performed with Pfu Ultra II polymerase (Agilent Technologies) and 1 µl antennal cDNA, according to manufacturer's instructions. AtraAOX2 5′ and 3′ regions were obtained by rapid amplification of cDNA ends (RACE). For 5′and 3′ RACE, antennal RACE cDNA was synthesized from 1 µg of male antennal total RNA at 42°C for 90 min using SMARTer™ RACE cDNA amplification kit and transcribed with SMARTScribe™ Reverse Transcriptase, 5′or 3′-CDS primer and SMARTer II A oligonucleotide (Clontech, Mountain View, CA). RACE PCR was performed with Advantage GC polymerase kit (Clontech), gene specific primers designed for high GC content (3′R-AtraAOX2: 5′-TCCGTCGTTGCGGCGGCGCGTTCGGC-3′; 5′R-AtraAOX2: 5′-CTCGACCAATCCGGTCGTGGCGCGCC-3′), universal primer mix (Clontech) and RACE antennal cDNA. Touchdown PCR was performed under the following amplification program: 94°C, 30 s to activate Advantage GC polymerase; followed by 5 cycles of two segment PCR at 94°C, 30 s and 72°C, 3 min, then 5 cycles of thee segment PCR at 94°C, 30 s; 70°C, 30 s and 72°C, 3 min and 40 cycles of 94°C, 30 s; 68°C, 30 s and 72°C for 30 min. Final extension was performed at 72°C for 6 min. 5′ and 3′ RACE AtraAOX2 amplicons were purified and sequenced (Davis Sequencing Inc). The full-length AtraAOX2 cDNA was finally amplified with Pfu Ultra II polymerase, using forward primer 5′-ATGGATCGCATAGTGTTTACTG-3′ and reverse primer 5′-TTATGGTGAGAAGACGAATTC-3′ and the following conditions: 94°C, 30 s to activate Advantage GC polymerase; followed by 40 cycles of 94°C, 30 s; 68°C, 30 s and 72°C for 30 min, and final extension was performed at 72°C for 6 min. The full-length DNA was cloned into pPCR-Script Amp SK + cloning vector (Agilent Technologies) and sequenced (Davis Sequencing Inc). All new data have been deposited in GenBank (KC952900).

### Distribution of AtraAOX2 in various tissues

*A. transitella* adults were dissected on ice using a stereo microscope (Zeiss, Germany). Tissue samples (antennae, legs, fat body, and heads) were collected and washed with PBS (140 mM NaCl, 27 mM KCl, 8 mM Na_2_HPO_4_, 1.5 mM KH_2_PO_4_, pH 7.4). Tissues were directly homogenized in 10 mM Tris-HCl (pH 8) without detergent using microtube pestle. The supernatant was removed, placed in new tubes by centrifugation for 10 min at 13,300 rpm and 4°C (Thermo, MicroCL 17R). Protein concentration was determined using a Bio-Rad Quick Start Bradford Dye Reagent (1×) in SmartSpec 3000 (Bio-Rad, Hercules, CA). 30 µg proteins of tissues were mixed in 5× native sample buffer lacking β-mercaptoethnol and sodium dodecyl sulfate (SDS) (final concentration 1×). Samples were subjected to 7.5% Native-PAGE gel in Laemmli's systems [59]. After electrophoresis on ice, the gel was fixed and stained with 0.1% Coomassie Brilliant Blue R-250. Another gel for enzyme activity (zymogram) was immersed in 0.1 M potassium phosphate buffer (pH 8) for 5 min, and then the activity band of aldehyde oxidase was developed with a mixture comprising the 0.1 M potassium phosphate buffer (pH 8), 0.1 mM phenazine methosulfate, 0.4 mM thiazolyl blue tetrazolium bromide (MTT), and 1 mM benzaldehyde as substrate at room temperature in the dark [56].

### Construction of recombinant baculovirus

In an initial attempt to express AtraAOX2 in a bacterial expression system, we cloned the ORF into the expression vector pET22-b(+) (EMD Chemicals, Gibbstown, NJ) and pQE-30 Xa (Qiagen, Valencia, CA), and attempted to express using BL21 (DE3) (EMD Chemicals, Gibbstown, NJ), M15[pREP4] (Qiagen), and TP-1000 (a gift from John Enemark's laboratory, University of Arizona) as host cells and with various IPTG concentrations and incubation temperatures following the manufacturer's instructions or Alfaro et al. [60].

For the baculovirus expression system, we cloned the ORF into pFastBac1, the following primers with restriction endonuclease sites (underlined) were designed: AtraAOX2PCR Forward (5′- GGATCCATGGATCGCATAGTGTTTACT-3′) and 6×histidine-appendaged (bold letters) or no 6×histidine-appendaged AtraAOX2PCR Reverse (5′-GGTACCTTA**ATGATGATGATGATGATG**TGGTGAGAAGACGAATTC-3′). PCR amplifications were performed with PfuUltra™ II Fusion HS DNA polymerase (Agilent Technologies) under the following condition: 94°C for 5 min, 33 cycles of 94°C for 30 s, 55°C for 40 s, 72°C for 3 min, and 72°C for 10 min. PCR products were cloned into PCR-Script Amp Cloning vector (Agilent Technologies) before being cloned into pFastBac1. Plasmids were extracted using the QIAprep spin mini prep kit (Qiagen) and sequenced using ABI 3730 automated DNA sequencer at Davis Sequencing. We followed Bac-to-Bac Baculovirus Expression System protocol (Invitrogen). pFastBac1 recombinant plasmid transformed into MAX efficiency DH 10Bac cells (containing bacmid and helper). Then, we isolated recombinant bacmid DNA containing AtraAOX2-His ORF. Recombinant bacmid DNA was transfected into inst Sf21 cells (Invitrogen) using Cellfectin II reagent (Invitrogen). We amplified recombinant baculovirus stock (5 × 10^7^ plaque-forming units (pfu)/ml) for further experiments.

### Recombinant protein expression and purification

Sf21 cell (1 × 10^7^ cells) was infected with viral stock at a multiplicity of infection (MOI) of 20 pfu/cell. After 5 h, fresh serum-free medium Sf-900 II SFM including co-factors (250 µl of trace element solution, 1 µg/ml riboflavin, and 50 µM sodium molybdate) was replaced [60]. After incubation at 27°C, cells were harvested at 3 days post-infection (p.i.). The harvested cells were mixed with washing buffer (50 mM NaH_2_PO_4_, 300 mM NaCl, 20 mM imidazole, pH 8), Insect PopCulture reagent (EMD Chemicals, Gibbstown, NJ), and protease inhibitor cocktail (1 µl/ml). The cell mixture was agitated using 20 gauge syringe. Cell lysates were isolated by centrifugation at 14000 rpm for 1 h at 4°C. Isolated cell lysates were combined with nickel-charged resin (Ni-NTA superflow, Qiagen) and incubated for 1 h by shaking at 200 rpm on ice. The suspension was loaded into a disposable polypropylene column (Thermo, Rockford, IL), washed twice with wash buffer (50 mM NaH_2_PO_4_, 300 mM NaCl, 20 mM imidazole or 50 mM imidazole, pH 8) and proteins were eluted with elution buffer (50 mM NaH_2_PO_4_, 300 mM NaCl, 100 mM, 150 mM, 200 mM, or 250 mM imidazole, pH 8) using gradient method (modified from Qiagen's manual). Elutes were desalted and concentrated by using Ultracel 100 K (100 kDa cut-off) (EMD Chemicals, Gibbstown, NJ). The protein concentration was determined with the Bio-Rad protein assay kit (Bio-Rad). Fractions and desalted samples were subjected to 7.5% SDS-PAGE (Bio-Rad). After electrophoresis, the gel was fixed and stained with 0.1% Coomassie Brilliant Blue R-250.

### Gel electrophoresis and staining

Reducing sample was prepared by adding reducing sample buffer containing β-ME and SDS and boiled for 5 min. Non-reducing sample was prepared without boiling by adding non-reducing sample buffer lacking βME but SDS was added. Reducing sample was subjected to 7.5% SDS-PAGE (Bio-Rad). After electrophoresis, the gel was fixed and stained with 0.1% Coomassie Brilliant Blue R-250. Non-reducing sample was subjected to 7.5% SDS-PAGE (Bio-Rad). After electrophoresis on ice, the gel was immersed in 0.1 M potassium phosphate buffer (pH 8) for 5 min, and then the activity band of AtraAOX2 was developed with a mixture comprising the 0.1 M potassium phosphate buffer (pH 8), 0.1 mM phenazine methosulfate (Sigma-Aldrich), 0.4 mM thiazolyl blue tetrazolium bromide (MTT) (Sigma-Aldrich), and 1 mM benzaldehyde as substrate at room temperature (RT) in the dark [56].

### SDS-PAGE and Western blotting analysis

Insect Sf21 cells (1 ×10^6^ cells) were infected in a 6-well plate (35-mm diameter well) at a multiplicity of infection (MOI) of 10 pfu/cell. After incubation at 27°C, the cells were harvested at 3 days post-injection. After centrifugation at 3,000 rpm for 10 min, cells infected with virus were washed twice with PBS and mixed with 100 µl of 2× protein sample buffer and boiled. The media were loaded in Ultracel 100 K and concentrated to 100 µl volume. The total cellular lysates of 10 µl (1 × 10^5^ cell-equivalent) and 10 µl media were subjected to 7.5% SDS-PAGE. After electrophoresis, gels were fixed and stained with 0.1% Coomassie Brilliant Blue R-250. For Western blotting analysis, the gel was transferred into a polyvinylidene fluoride (PVDF) membrane at 30 V overnight on ice. After transferring, the PVDF membrane was blocked in TBST-3% skim milk (Tris-Buffered Saline and Tween 20) for 1 h and then was incubated with Anti-6×Histidine primary antibody (1∶10,000) (Sigma-Aldrich) in TBST-3% skim milk for 1 h at RT. The membrane was washed twice with TBST for 5 min in a rotary shaker and incubated with horseradish peroxidase (HP)-conjugate sondary antibody (1∶10,000) (Sigma-Aldrich) in TBST-3% skim milk in 1 h at RT. The membrane was washed twice with TBST for 10 min in a rotary shaker and then incubated with chemoluminescent substrate for 5 min (ECL Plus Western Detection Kit, GE Healthcare), and film was developed in a dark room.

### Determination of enzyme activity

The thermal stability of AtraAOX2 was measured by pre-incubating the enzyme for 10 min at different temperatures (20–90°C) before initiating the reaction with a substrate solution (0.1 M potassium phosphate buffer, pH 8, 0.1 mM phenazine methosulfate, 0.4 mM MTT, and 1 mM benzaldehyde). For heat inactivation, AtraAOX2 was preheated at 60°C without a substrate. Samples were removed at 10 min intervals for 70 min and chilled on ice. Enzyme activity was determined at 30°C for 30 min using a substrate solution, and then quenched with 10% acetic acid. The effect of temperature on AtraAOX2 activity was evaluated based on the purple insoluble MTT formazan formation. To determine the optimum pH and active buffer of AtrAOX2, the enzyme activity was measured at various pH values (4–8) or buffers (pH 8, potassium phosphate buffer; pH 8, Tris–HCl buffer; pH 8, sodium phosphate). Enzyme activity was performed as described above. The formation of insoluble MTT formazan was measured at 570 nm. All experiments were performed in triplicate.

### Activity on volatile aldehydes and inhibition assay

The activity of AtraAOX2 (1 µg) on various aldehydes was determined at 30°C for 1 h and then quenched with 10% acetic acid. The enzyme activity was measured as described above. Substrates lacking AtraAOX2 were used as blank control in order to prevent nonenzymatic background activity. Recombinant AtraAOX2 was subjected to 7.5% Native-PAGE gel. AtraAOX2 activity assay was performed as described above using various plant volatile aldehydes and aldehyde-containing pesticides as substrates. The effects of various inhibitors on AtraAOX2 were determined as described previously [13], [56]. AtraAOX2 (1 µg) was preincubated with various inhibitors for 3 min at 30°C without a substrate. The activity was assayed with substrate solution (0.1 M potassium phosphate buffer, pH 8, 0.1 mM phenazine methosulfate, 0.4 mM MTT, and 2 mM propanal). The mixture was incubated at 30°C for 1 h and quenched with 10% acetic acid. The enzyme activity was measured as described above. All experiments were performed in triplicate.

### Chemicals

Hydroquinone, sodium deoxycholate, Triton X-100, potassium cyanide (KCN), isopropanol, ethanol, β-estradiol, p-hydroxymercuribenzoate, sodium azide (NaN_3_), antimycin A, dinitrophenol, quinacrine, methanol, β-ecdysone. benzaldehyde, propanal, hexanal, heptanal, nonanal, trans-2-hexenal, trans-2-nonanal, citral, vanillin, xanthin, acetaldehyde, and acrolein were purchased from Sigma-Ardrich. Rotenone, digitonin, menadione and aldicarb were gifts from Dr. Bruce Hammock. Z11Z13–16Ald was synthesized as previously described [12].
